# Social Media for Public Health: Framework for Social Media–Based Public Health Campaigns

**DOI:** 10.2196/42179

**Published:** 2022-12-14

**Authors:** Isabella de Vere Hunt, Eleni Linos

**Affiliations:** 1 Program for Clinical Research and Technology Stanford University Palo Alto, CA United States; 2 Nuffield Department of Primary Care Health Sciences University of Oxford Oxford United Kingdom

**Keywords:** social media, digital heath, health communication, campaign, public health, framework, health promotion, public awareness, misinformation, tailored message, tailored messaging, information sharing, information exchange, advertise, advertising

## Abstract

The pervasiveness of social media is irrefutable, with 72% of adults reporting using at least one social media platform and an average daily usage of 2 hours. Social media has been shown to influence health-related behaviors, and it offers a powerful tool through which we can rapidly reach large segments of the population with tailored health messaging. However, despite increasing interest in using social media for dissemination of public health messaging and research exploring the dangers of misinformation on social media, the specifics of how public health practitioners can effectively use social media for health promotion are not well described. In this viewpoint, we propose a novel framework with the following 5 key principles to guide the use of social media for public health campaigns: (1) tailoring messages and targeting them to specific populations—this may include targeting messages to specific populations based on age, sex, or language spoken; interests; or geotargeting messages at state, city, or zip code level; (2) including members of the target population in message development—messages should be designed with and approved by members of the community they are designed to reach, to ensure cultural sensitivity and trust-building; (3) identifying and addressing misinformation—public health practitioners can directly address misinformation through myth-busting messages, in which false claims are highlighted and explained and accurate information reiterated; (4) leveraging information sharing—when designing messages for social media, it is crucial to consider their “shareability,” and consider partnering with social media influencers who are trusted messengers among their online followers; and (5) evaluating impact by measuring real-world outcomes, for example measuring foot traffic data. Leveraging social media to deliver public health campaigns enables us to capitalize on sophisticated for-profit advertising techniques to disseminate tailored messaging directly to communities that need it most, with a precision far beyond the reaches of conventional mass media. We call for the Centers for Disease Control and Prevention as well as state and local public health agencies to continue to optimize and rigorously evaluate the use of social media for health promotion.

## Introduction

The pervasiveness of social media is irrefutable; 72% of adults and 84% of adults aged 18-29 years report use of at least one type of social media platform and an average daily usage of 2 hours [1,2]. Social media has been shown to influence public opinion, political views, and purchasing behaviors, as well as health-related behaviors such as diet and exercise [3-6]. This offers a powerful tool through which we can rapidly reach large segments of the population with tailored health messaging. It has also become a potent arena for widespread dissemination of misinformation and disinformation, posing its own public health threat [7-9]. However, although numerous studies have explored the dangers of misinformation and have used data from social media to interpret public attitudes [10], the specifics of how public health practitioners can effectively use social media for health promotion are not described. In this viewpoint, we propose a new framework with the following 5 key principles to guide the use of social media for public health campaigns: (1) tailoring messages and targeting them to specific populations; (2) including members of the target population in message development; (3) identifying and addressing misinformation; (4) leveraging information sharing; and (5) evaluating impact by measuring real-world outcomes.

## Tailoring Messages and Targeting Them to Specific Populations

In addition to connecting individuals, social media platforms, including Facebook, Instagram, Twitter, and TikTok, have sophisticated advertising platforms that facilitate targeting of advertisements to specified populations with far greater precision than conventional mass media. For example, a multivitamin company can send advertisements to women aged 25-40 years living in Manhattan with a household income in the top 10% in the United States and an interest in organic food. Researchers have used these targeted advertising tools for the purpose of study recruitment [11]. For example, Reiter et al [12] used Facebook’s targeted advertising to recruit young gay and bisexual men for a human papillomavirus vaccination intervention by selecting for English-speaking males in the United States aged 18-25 years with any of the following “interest” filters: bisexuality; homosexuality; same-sex relationship; genderqueer; gay pride; lesbian, gay, bisexual, and transgender (LGBT) community; LGBT culture; or rainbow (LGBT movement) [12]. Public health practitioners can leverage ad targeting to send tailored health messages to specific populations based on age, sex, or language spoken; interests, such as “smoking,” “aerobic exercise,” or “McDonalds”; or geotarget messages at state, city, or zip code level. Targeting messages by language spoken is particularly relevant to immigrant and refugee communities, for whom language barriers may limit understanding of alternative sources of health information; for example, in Germany, social media advertisements in migrants’ languages of origin increased COVID-19 vaccine appointment bookings by 133% for Arabic speakers and 76% for Russian speakers [13]. Of note, Facebook continually reviews the available ad targeting options and ad controls with the aim of reducing the possibility of ad discrimination; for example, in January 2022 they removed previously available detailed targeting options that “relate to topics people may perceive as sensitive,” which include health causes such as “lung cancer awareness” and “chemotherapy” [14]. We therefore recommend frequent monitoring of targeting options when planning campaign implementation to ascertain what will be available for use.

A key challenge in using social media for public health is that algorithms are designed to present advertisements a person is likely to agree or engage with [15], but in public health, we often seek to reach those who disagree, for example, convincing a smoker to quit or a reluctant parent to vaccinate their child. One way to address this is to tailor messages and separate them into narrower ad sets for specific populations. For instance, although COVID-19 vaccine promotion messages might be unpopular among vaccine-hesitant groups, we can increase message salience by tailoring them to subsets of the target population—a message debunking fertility concerns could be sent to women aged 25-30 years with an interest in “motherhood”; a video by a Spanish-speaking doctor could be delivered to Spanish-speaking adults in a zip code area with low vaccination rates; and a video by a Methodist priest could be sent to people interested in the “Methodist church” [16]. The ability to rapidly pilot-test multiple iterations can identify the most engaging messages for each group. By structuring campaigns into ad sets, we can also allocate more budget to populations who need it most; for example, using indices such as the California Health Place Index, we can preferentially allocate funds to lower health index zip code areas. An et al [11] propose a useful precision public health campaign framework to guide the use of targeted advertising tools on social media to deliver tailored health messages to particular population segments.

## Including Members of the Target Population in Message Development

Engaging community partners when designing public health messaging is paramount in building trust and ensuring effectiveness. Messages should be designed with and approved by members of the community they are designed to reach, to ensure cultural sensitivity and trust-building. One way to achieve this is to assemble an advisory board, including members of the target population, and reflecting the demographics of users of the intended platform. For example, approximately 43% of TikTok’s audience is 18-24 years of age, and only 3% is aged >55 years [17]; thus, input from younger, Gen Z voices would be crucial for a campaign running on TikTok. Further, any ad targeting strategies should be transparent and sensitive to the potential for discriminatory ad targeting. Indeed, journalists have demonstrated, with historical ad targeting options available on Facebook, how easy it would be to exclude users whom Facebook classifies as a member of a racial or ethnic minority group from target audiences [18]. Although race categories have subsequently been removed from explicit targeting options on Facebook, the ability to direct ads to specific racial groups is still implicitly possible (to varying degrees of accuracy) via proxies such as zip code targeting. Therefore, we call for discussion with and approval by an advisory board of any proposed ad targeting strategies, alongside a clearly documented rationale that aims to benefit the target audience, prior to campaign implementation. When translating messages, using input from native speakers to ensure optimal language choice rather than relying on automated translations is crucial.

## Identifying and Addressing Misinformation

Misinformation on social media has been shown to influence health attitudes; in a randomized controlled trial assessing the effect of web-based misinformation on COVID-19 vaccine intentions, recent exposure to misinformation decreased vaccine intent by 6.4% among participants who previously stated they would definitely accept a vaccine [19]. The extent to which social media facilitates dissemination of misinformation was exemplified by infodemics—defined by the World Health Organization as an overabundance of both inaccurate and accurate information—during the COVID-19 pandemic [20,21]. Vosoughi et al [8] hypothesize that false news reaches more people than the truth does because it has a higher degree of novelty and provokes stronger emotional reactions of recipients, making it more likely to be passed on. Public health practitioners can directly address misinformation through myth-busting messages, in which false claims are highlighted and explained and accurate information reiterated. This should be an iterative process, beginning with message design and continuing through active comment moderation, including direct responses to false comments during a live campaign. In a randomized controlled trial of messages debunking highly prevalent health information in Sierra Leone, direct and detailed debunking was most effective [22]. Live interactions are a key part of how information is disseminated on social media, yet traditional mass media communication models do not account for this interactivity. Parackal et al [23] propose the dynamic transactional model of communication as a suitable framework for modelling the “two-way communication” in which both the sender and the receiver actively participate in the communication process that takes place on social media [23].

## Leveraging Information Sharing by the Target Population

Trusted messengers, including healthcare providers, religious leaders, and celebrities, can play an important role in public health messaging; for example, basketball player Magic Johnson’s announcement of his HIV-positive status in 1991 was correlated with increased condom use among Black and Hispanic individuals [24]. Yet social media differs from traditional broadcast media in the rapidity at which messages disseminated from an original source can be publicly reshared by the target population. In a survey experiment on Facebook of 1489 adults, 51% reported that a health article on diabetes was well reported and trustworthy when it was shared by a public figure they trusted, whereas only 34% thought the same article was trustworthy when it was shared by someone they did not trust [25]. When designing messages for social media, it is crucial to consider their “shareability”—can the message be designed in a way that encourages users to share it with their friends? Partnering with social media influencers—users of social media with established credibility among their followers—is a useful approach for leveraging trusted messengers.

## Evaluating Impact by Measuring Real-world Outcomes

Evaluation of social media–based public health campaigns should include measurement of the health-related behavior of interest in the target population. Breza et al [26] provide an excellent example, as follows: in a cluster randomized controlled trial, investigators used distance travelled in treatment regions, measured using mobile phone location data of Facebook users, as well as COVID-19 infections recorded at the zip code level, as the outcome measures to assess the impact of a social media advertising campaign asking participants to avoid holiday travel to reduce COVID-19 infections. In our own work, we have piloted an approach using analysis of foot traffic data to tanning salons as an outcome measure to assess the impact of a social media campaign aiming to reduce indoor tanning. Social media platforms also record web-based evaluation metrics, including number of people reached, average duration of videos viewed, reactions, shares, unique link clicks, and cost per individual reached. How these web-based metrics map onto real-world behaviors is unclear; reporting of web-based outcome measures alongside real-world measures can improve our understanding of how these metrics correlate with health-related behavioral change.

## Conclusions

Leveraging social media to deliver public health campaigns enables us to capitalize on sophisticated for-profit advertising techniques to disseminate tailored messaging directly to communities that need it most, with a precision far beyond the reaches of conventional mass media. We do not present social media as a public health panacea; grave concerns about cyberbullying, privacy breaches, and misinformation on social media must be addressed in parallel [27]. Further, collaboration between public health scientists and technology companies will be vital to support widespread and potentially expensive ad campaigns, with the success of such partnerships highlighted by extensive COVID-19 vaccine promotion efforts supported by Facebook ad credits [16]. However, in a nation in which three-quarters of adults use social media, for some of whom social media will be the only source of health information, the Centers for Disease Control and Prevention as well as state and local public health agencies must optimize and rigorously evaluate its use for health promotion.

## Abbreviations

LGBT: lesbian, gay, bisexual, and transgender
